# Coupling Microfluidic Platforms, Microfabrication, and Tissue Engineered Scaffolds to Investigate Tumor Cells Mechanobiology

**DOI:** 10.3390/mi10060418

**Published:** 2019-06-22

**Authors:** Martial Millet, Raoua Ben Messaoud, Carole Luthold, Francois Bordeleau

**Affiliations:** CHU de Québec-Université Laval Research Center (Oncology division), Université Laval Cancer Research Center and Faculty of Medicine, Université Laval, Québec, QC G1R 3S3, Canada; martial.millet.1@ulaval.ca (M.M.); raoua.ben-messaoud.1@ulaval.ca (R.B.M.); carole.luthold.1@ulaval.ca (C.L.)

**Keywords:** tumor microenvironment, mechanobiology, microfluidic, microfabrication, extracellular matrix, tumor stiffness, tumor engineered models, disease modeling

## Abstract

The tumor microenvironment (TME) is composed of dynamic and complex networks composed of matrix substrates, extracellular matrix (ECM), non-malignant cells, and tumor cells. The TME is in constant evolution during the disease progression, most notably through gradual stiffening of the stroma. Within the tumor, increased ECM stiffness drives tumor growth and metastatic events. However, classic in vitro strategies to study the TME in cancer lack the complexity to fully replicate the TME. The quest to understand how the mechanical, geometrical, and biochemical environment of cells impacts their behavior and fate has been a major force driving the recent development of new technologies in cell biology research. Despite rapid advances in this field, many challenges remain in order to bridge the gap between the classical culture dish and the biological reality of actual tissue. Microfabrication coupled with microfluidic approaches aim to engineer the actual complexity of the TME. Moreover, TME bioengineering allows artificial modulations with single or multiple cues to study different phenomena occurring in vivo. Some innovative cutting-edge tools and new microfluidic approaches could have an important impact on the fields of biology and medicine by bringing deeper understanding of the TME, cell behavior, and drug effects.

## 1. Introduction

**Figure 1:**
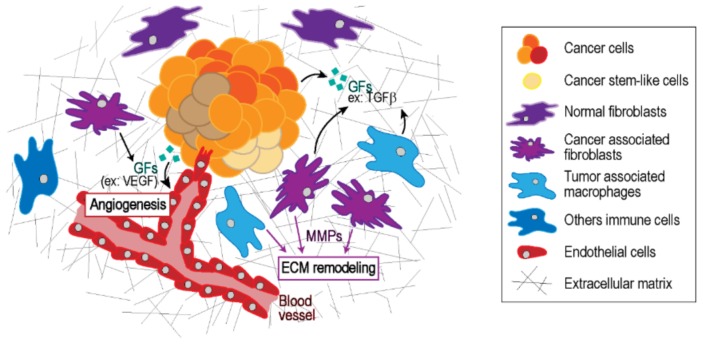
The tumor microenvironment representation. The tumor microenvironment (TME) is a considerably heterogeneous niche and consists of proliferating tumor cells, infiltrating immune cells, the surrounding extracellular matrix, blood vessels, and a variety of associated stromal cells. Intercellular communication within this niche is driven by a complex and dynamic network of soluble proteins. In solid tumors, these soluble proteins are synthesized by local tumor and stromal cells, including growth factors, angiogenic factors, extracellular matrix (ECM) remodeling proteins, chemokines and interleukins, and enhance tumor cell proliferation and invasion and inhibit tumor cell apoptosis.

A tumor mass contains a heterogeneous population of cancer cells and infiltrating host cells such as endothelial cells, immune cells, fibroblasts, pericytes, adipocytes, or stem cells (Figure 1). Some types of tumor microenvironment (TME) cells can secrete factors and produce extracellular matrix (ECM) proteins which interact directly or indirectly with cancer cells. The mechanical and physical properties of the tumor microenvironment are increasingly appreciated as a key regulator of biological functions such as tumor growth, metastasis, and drug resistance. Accordingly, state-of-the-art microfabrication and microfluidic approaches have been developed to help uncover the underlying biological processes of cancer-associated mechanobiology.

In order to study crosstalk between cancer cells and TME in humans, bioengineers are using microfabrication and tissue-engineering microfluidics to biomimic TME. The aim is to reconstitute the architecture, the physical and chemical conditions, and the functional microsystem within a given tissue [1,2]. Moreover, heterogeneity of different cancer cell populations and the diversity of TME-associated cells constitute one of the major challenges for recreating the TME artificially. The heterogeneous architecture and anisotropic nature of the tumor ECM in vivo is another challenging aspect to recreating the TME. The main strategy is to control every condition and the whole infrastructure of reconstituted tissue to investigate dynamics of cancer development and therapeutic approaches coupled with constant perfusions of nutrients with a possible short-term analysis [3].

Nowadays, cancer research and associated drug study are mostly conducted using two-dimensional and three-dimensional in vitro cell cultures and also animal models in vivo. 3D cell culture is getting slightly closer to understanding TME than the 2D monolayer harvesting method. 3D cell culture mimics basic biostructure and some ECM biomechanical and physiological properties, and also various parameters of TME can be modulated such as nutrient gradients and oxygen level [4,5,6,7]. In addition to the complication of microenvironment modeling, the limits of these techniques can be demonstrated through the gap between the success of many drugs in preclinical in vitro analysis and their breakdown in the patients. The study of animal models in vivo is time-consuming, expensive, inconsistently repeatable, and poses ethical issues [7]. The main advantage of this model is to work on a complete tumor microenvironment even if it does not reflect entirely tumor development in humans. Moreover, some specific models as immunosuppressed mice poorly mimic actual phenomena occurring in TME cells because immune cells play a primordial role in both pro- and anti-tumor responses [8].

Here, we reviewed the main features of the TME, ranging from the mechanical property alteration linked to tumor progression, mechanobiology of the cell response to soluble factors, as well as the different cellular contributor to the TME. We described the roles, advantages, and perspectives of applied microfabrication and tissue-engineered microfluidics that aim at recreating the complexity of the TME.

## 2. Tumor Microenvironment

Initiation, progression, and metastasis are the three stages of tumorigenesis which are influenced by the ECM and the overall biochemical state of the TME. The ECM provides architecture, elasticity, and strength to tissues. However, the ECM structure and chemical composition are regulated by a number of cell-mediated and enzyme-mediated processes. The actual ECM biochemical composition and biomechanical complexity are both tissue specific and linked to tumor progression [8,9]. Stromal enzymes irreversibly regulate ECM architecture crosslinking collagen and elastin fibers such as lysyl oxidase (LOX) and transglutaminase 2 (TG2) from which cells can reorient and cross-link collagen and elastin fibers [10,11]. The crosslinking of ECM components is largely responsible for the increased stiffness of the TME [12]. In addition, tumor associated macrophages (TAMs) and cancer associated fibroblasts (CAFs) secrete matrix metalloproteases (MMPs) as well as heparanase-promoting cathepsin L that act as ECM degrading protease, which in turn favor metastasis, angiogenesis, and inflammation [13,14]. Furthermore, CAFs, and to a lesser extent tumor cells, can secrete new ECM components and actively participate in remodeling the ECM architecture [15].

The tumor-mediated increase of ECM stiffness deregulates stromal homeostasis providing an acidic, hypoxic, or low-nutrient environment. At the same time, the stiffer ECM offers greater survival rate to tumor cells [8]. In fact, ECM stiffness plays a role in driving malignancy and influences the switch of stromal cell phenotypes [11,16,17,18]. Exact composition of tumor stroma fluctuates according to tumor type and tissue location, but the most abundant components of the ECM are collagen, fibronectin, laminin, proteoglycan, and hyaluronan [8,13,19]. Integrins are the main family of cell surface receptors which interact with ECM fibrils promoting cell adhesion, cell migration, cell proliferation, and cell survival [20]. The presence of specific integrins at the cell surface control to which ECM component the cells are able to attach [21]. In turn, ECM stiffness sustains tumorigenesis by assembling integrin-mediated focal adhesion (FA) complexes and stimulating integrin-dependent mechanotransduction [17,22,23]. Once activated, it promotes focal adhesion kinase (FAK)-associated pathways, cytoskeletal remodeling, and cellular contractility [11,20,24,25]. 

The mechanical properties and composition of the ECM also influence how soluble factors contribute to tumor progression. In vivo, growth factors (GFs) can be found in either soluble form or bound to the ECM, including to structural proteins, such as collagen, and glycosaminoglycans [26]. ECM-bound GFs are more efficient to induce enhanced downstream responses with lower doses. This phenomenon is explainable by higher local concentration of ECM-bound GFs and covalent tethering of GFs to the ECM. With ECM-bound GFs, integrins can be clustered to growth factor receptors (GFRs) and enhance signaling by synergistic responses [26]. Interestingly, several signaling pathways triggered by chemokines, cytokines, and GFs, and which are known to induce cell growth and migration, are in fact modulated by cell contractility and ECM stiffness [13,14,27,28,29,30]. For instance, ECM stiffness-mediated cell contractility increases endothelial cell response to vascular endothelial growth factor (VEGF), which in turn can promote tumor associated angiogenesis and vessel permeability [12,31]. Alternatively, cell contractility can also increase the availability of otherwise unavailable GFs. For example, transforming growth factor β (TGFβ) is usually found to be encapsulated within the latency-associated peptide (LAP) bound to the ECM. When cells exert sufficient forces on a LAP complex bound to a stiff ECM, it will distort and unwrap causing the release of TGFβ in the TME [32]. Taken together, these data provide good examples on how ECM stiffness and cell contractility influence cellular responses to GFs but also partially affect GFs availability in the TME.

The TME also contains several types of stromal cells that interact with the ECM while playing an important role in supporting the tumor. First, malignant cells, myeloid cells, and CAFs produce angiogenic factors and stimulate sprouting of vascular endothelial cells [33]. Pericytes support vascular endothelial cells in neoangiogenesis [34]. In parallel, tumor-cell-produced VEGFC and VEGFD stimulate neogenesis of lymphatic vessels [35]. Then, perpetual inflammation state, wound healing features, and cancer cells attract different kinds of infiltrating immune cells. Metelitsa et al. showed that C-C Motif Chemokine Ligand 2 (CCL2)-producing neuroblastoma cell lines allow the infiltration of natural killer T cells (NKTs) [36]. B lymphocyte subtypes B_10_ and B_reg_, tumor-associated macrophages (TAMs), and myeloid-derived suppressor cells (MDSCs) produce immunosuppressive factors such as interleukin 10 (IL-10) and interleukin 12 (IL-12), which are used by malignant cells to hide from immunosurveillance [37,38]. Moreover, MDSCs are known to trigger tumor-promoting phenotypes of TAMs [39]. Thirdly, adipocytes secrete adipokines, which promote cell survival [40], angiogenesis, and inflammation [41], but also provide fatty acids to the TME, fueling cell proliferation [42]. Finally, while transformation of fibroblasts into CAFs can be mediated by TGFβ, microvesicles released by cancer cells can also induce fibroblast activation into CAF [43]. Moreover, this microvesicle-mediated CAF transformation also depends on the underlaying ECM stiffness [44]. Considering that all the different cell types within the TME contribute to tumor development [16,22], it is important to address cell‒cell and TME‒cell interactions in relevant tissue engineered models to fully grasp how tumor mechanical properties drive disease progression.

## 3. Use of Microfabrication and Microfluidic Systems to Engineer the Tumor Microenvironment (TME)

Nowadays, animal models are still considered as the gold standard. However, controlling the TME in mouse models is inherently challenging due to the complexity, with few tools allowing perfect controls of TME components such as ECM stiffness or availability of soluble factors [45]. The alternative classical cell culture approaches offer a simplified system, which in turn lack the complexity observed in the disease. Historically, most findings obtained using classical approaches fail to translate into applicable treatments [46]. Thus, a major challenge to study the role of the TME in tumor progression is our ability to accurately mimic the in vivo environment in vitro. In this context, several new approaches have been adopted over the years in order to thwart the simplicity of plastic dish cell culture while allowing the precise control of biochemical and mechanical parameters not possible in animal models.

### 3.1. 2D Microfabricated Substrate

**Figure 2:**
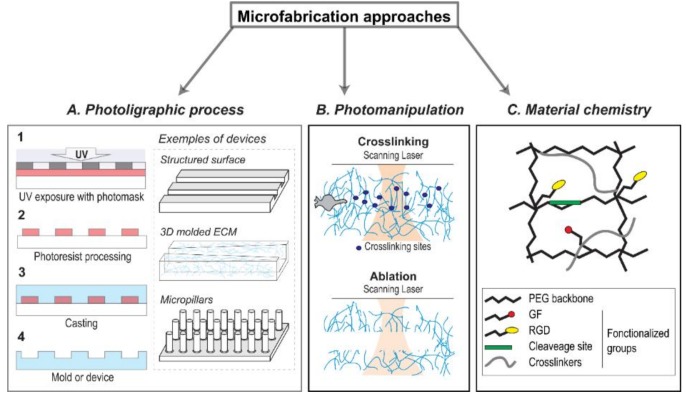
Illustration of different microfabrication approaches to mimic in vitro 3D microenvironment. Multiple methods, involving either natural or synthetic biomaterials, have been developed to engineer extracellular matrix (ECM) scaffolds and recapitulate the 3D physical features of the in vivo native ECM. (**A**) Simplified workflow for photolithography-based microfabrication where the first step is to inscribe the desired pattern present on a photomask in a photoresist. UV exposed photoresist can then be developed to obtain either a functional device or a negative mold that can then be used to cast a device from the desired material. This ubiquitous technique is routinely used to generate microstructured surfaces for cell adhesion, mold complex 3D scaffolds and microfluidic devices, or advanced active force sensors such as the micropillar systems. Furtermore, to increase collagen hydrogel stiffness used for cell culture and achieve stiffness levels closer to those found in tumor tissues, collagen can be crosslinked (**B**) by using enzymatic (i.e., LOX, TG2) and non-enzymatic (i.e., glycation) collagen crosslinking but also photoactivable crosslinkers (i.e., riboflavin). Alternatively, laser photo-ablation can be used to write microstructures within 3D hydrogels scaffolds. Synthetic biomaterials (**C**), such as polyethylene glycol (PEG) hydrogel, are synthesized to incorporate different functional groups, e.g., the Arg-Gly-Asp (RGD) peptide sequences (cell binding sites) or MMP-sensitive peptides (MMP-degradable region) as part of their design.

The simplest implementation of this idea of tuning an in vitro model to mimic the in vivo condition is achieved using 2D microfabrication techniques. Cell attachment is dependent on ECM components such as fibronectin, collagen, or laminin, which allow, for instance, integrin signaling pathways activation [47]. To better understand how the spatial organization of these ECM components influences cell adhesion and spreading, several groups have relied on photolithography microfabrication [48], a high spatial resolution photoprinting process [49,50], which allows the transfer of ECM features of controlled shapes and sizes to a surface or even the creation of nano- and micro-structured patterns (Figure 2A). 

Using these systems, it was shown that cell contact surface area influences the cell cytoskeleton dynamic and mechanical properties [49,51]. The shape and geometry of the contact area also influence mechanical strain distribution within the cell [49,52]. Pushing this idea further, Kassianidou et al. generated a pattern that mimics the discrete distribution of ECM components seen in 3D on a 2D substrate and demonstrated that the initial position of the cell relative to the geometry of the available ECM regulates the actin fiber dynamic and organization, thus providing an “adhesion memory” to the cell [50,52]. Moreover, studies performed on several types of cancer cells showed that substrate stiffness and the inherent behavior of tumor cells are linked [11,17,29,53]. However, the TME mechanical properties display a large degree of spatial heterogeneities [54]. There is therefore a need to develop methods that allow the control of substrate. Work performed with engineered photosensitive materials have allowed researchers to generate substrates for which stiffness can be tuned in real time [55,56,57,58]. Interestingly, these substrates can allow fine spatial control of the substrate stiffness to study biological functions such as cell migration or cell fate [57,58]. Furthermore, several of these methods are flexible enough that they can be combined [58], enabling spatial and temporal control over substrate adhesion geometry and mechanical properties. These improvements are critical to achieve better in vitro tumor model design.

### 3.2. 3D Substrate—Engineered Extracellular Matrix (ECM) Scaffolds 

**Figure 3:**
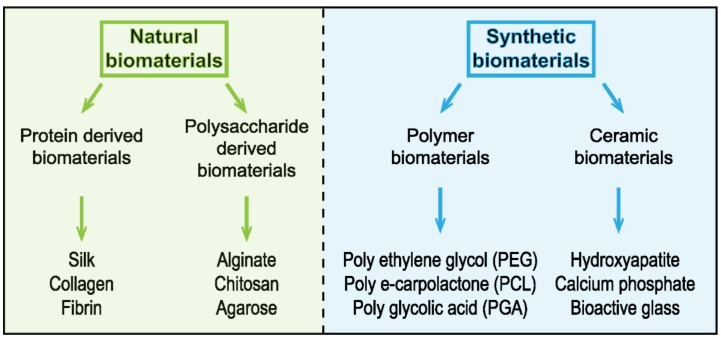
Classification of different biomaterials used as substrate for cell modeling.

Mimicking 3D microenvironment and ECM is one of the most challenging studies in order to explore the complexity of the in vivo system. Thus, it is really important to construct the closest 3D in vitro system to the native ECM [59]. The field of mechanobiology has focused on strategies involving either natural or synthetic biomaterials to attempt to recapitulate the 3D physical features of the in vivo environment (Figure 3). 

Researchers have used physiologically relevant biomaterials such as collagen [46]. Notably, collagen is one of the most abundant proteins of the ECM and contributes to increased tumor stiffness [13]. Collagen is known to self-assemble into a fibrous network, and it is possible to control its organization to preferentially obtain long and aligned fibers or very densely packed short fibers [60,61,62]. Collagen 3D architecture and density are known to influence cell migration parameters, including cell speed and migration persistence [60]. However, the stiffness of the typical collagen hydrogels used for cell culture are usually significantly lower than the tissue or tumor they are intended to recapitulate, ranging from around 50 Pa at a concentration of 1 mg/mL to 1 kPa at 10 mg/mL [12]. Strategies employed to overcome this technical issue have relied on using either enzymatic or non-enzymatic collagen crosslinking to achieve stiffness levels closer to those found in tumor tissues [63]. For example, non-enzymatic glycation can be used to crosslink collagen either in solution prior to polymerization or once the scaffold is established, thus providing a way to tune the stiffness of a collagen scaffold independently of its concentration and 3D architecture [64] (Table 1).

We and other groups have taken advantage of this approach as a way to model a tumor mechanical property to investigate the effects of 3D matrix stiffness-mediated angiogenesis, cancer cell contractility, and invasive properties [28,65,66]. Alternatively, photoactivable crosslinkers, such as riboflavin, can also be used to crosslink collagen [67,68] (Figure 2B). While such an approach theoretically allows the stiffness of collagen or any 3D ECM-based scaffolds to be precisely and spatially tuned, it has not seen widespread use in the field. This is likely related to the phototoxicity induced by the blue and UV wavelength needed for the photocrosslinking process [69]. Another method for controlling stiffness is to use recombinant versions of the enzymes that are responsible for the crosslinking of collagen scaffolds in vivo. Indeed, both the LOX and TG2 (transglutaminase 2) enzyme have been used to stiffen collagen scaffolds [70,71]. Other materials have been used such as fibrin or matrigel depending on its characteristics and the cancer research applications. For example, matrigel, characterized by its cytocompatibility and its tunable properties, was used in breast cancer modeling and helped to understand the role of cell assembly and progression in the development of the disease [72].

Other biomaterials have been considered as 3D scaffolds, notably the *Antheraea Mylitta* fibroin protein, a silk protein generated from the tasar silkworm [73] This silk-derived scaffold was used as an ECM equivalent for an in vitro breast cancer model [74]. Silk fibroin demonstrates promising mechanical properties such as stiffness strength [75]. It is characterized by its biocompatibility, biodegradability, high versatility, and a porous structure (∼50–500 μm pore size) [76]. Owing to its characteristics, silk fibroin fibers have been used in engineering and medicine. It is used as matrices for 3D cell culture, 3D microenvironment for cell attachment, growth, and co-culture for tumor cells [76]. The high porosity of the fiber plays an essential role in cell growth as it allows nutrient and waste exchanges [74]. Recently, it has been used in modeling different types of cancers, including hepatocarcinoma, osteosarcoma, breast, and prostate cancer [73,77,78]. Engineered scaffolds produced from natural biomaterials suffer from several intrinsic limitations, notably because of their heterogeneous and anisotropic nature [79]. In this context, synthetic biomaterials are increasingly used in biomedical research. The advantages of synthetic biomaterials include tunable chemistry, easy to control biomechanical properties, and biocompatibility and biodegradability [80,81,82]. Synthetic biomaterials can be synthesized to incorporate different functional groups within as part of their design, such as cell binding sites (e.g., the RGD peptide sequences) or the MMP-degradable regions (e.g., GGGPQGIWGQGK (PQ) peptide), resulting in physiologically relevant cell‒matrix interactions [83] (Figure 2C). Use of polyethylene glycol (PEG) hydrogel-containing RGD and MMP-sensitive peptides have helped highlight the growth of epithelial ovarian cancer cells [84]. Notwithstanding that matrixes composed of synthetic polymers present some disadvantages such as cytotoxicity and lower degradability [85], synthetic polymers offer the highest levels of control over their tailored chemistry and overall physical properties.

### 3.3. 3D Microfabricated Substrate 

To further control and design tunable 3D scaffolds, several groups have adopted molded microfabrication techniques based on cast PDMS (polydimethylsiloxane) or native ECM [86,87,88] (Table 2). 

In brief, the fabrication technique uses a photolithography process to obtain a master mold that is then used to cast a device containing desirable features. The main goal is to replicate the architecture and features such as density, pore size, and fiber alignment that are observed within the tumor stroma [89]. Results showed that channels with limiting cross sections similar to the space found in vivo act as a limiter for efficient cell migration [90]. Interestingly, use of microfabricated constriction has highlighted a previously unknown mechanism: the nucleus can rupture and generate DNA break when tumor cells migrate through severely limiting constrictions, an outcome that was further confirmed in vivo [91]. Alternatively, approaches have been developed to better mimic in vivo physiological conditions, and microfabrication techniques were applied to several biomaterials, including native ECM, proteoglycans, or polysaccharides in the stroma microarchitecture. For instance, microtracks can be manufactured from 3D collagen scaffolds as a physiologically relevant mimic of the collagen structure found within in vivo tumors [92]. Alternatively, others have used laser photo-ablation to write microstructures within hydrogels [93,94], which were subsequently used to study collective cell migration and MMP-independent tumor cell migration [95]. Combining some of the techniques that were originally designed as 2D system to generate 3D architecture was a great step toward obtaining better in vitro systems that more closely mimic in vivo conditions (Table 3).

### 3.4. Microfabrication for Force Measurement 

Fabricating an engineered TME is one step forward. However, the physical properties of the material used can directly influence cells. In fact, cellular response and adaptation to the physical environment is an active process, where cells continuously probe and exert force on their surrounding ECM. In turn, the ECM also provides active (traction, compression) or passive (stiffness, viscosity, plasticity) mechanical cues to the cells. While this interplay provides important cues that drive biological functions, such as cell migration and morphogenesis, it is entirely dependent on the generation of intracellular mechanical forces by the actomyosin contractile structures [96,97]. There are therefore increasing efforts being made in trying to implement force measurement as a part of microfabricated devices. By pushing the boundaries of microfabrication techniques, several groups have come up with strategies to manufacture devices that allow force measurements while mimicking the physical properties of the microenvironment as closely as possible. Microelectromechanical systems (MEMS) were developed to investigate cellular behaviors in the system, as well as MEMS sensors able to measure micronewton levels of forces or less while following the migration of a single cell. Many microdevices have been functionalized and tested to measure 3D cell forces, such as large cantilevers fabricated by soft lithography [98]. A mimetic tissue is capable of self-assembly between two cantilevers in a culture chamber. The deflection of the pillars over time allows fast and automated detection of the bulk contraction of the tissue [99]. An implementation of this system with multiple anchoring pillars was able to demonstrate how mechanical strain, cellular forces, and matrix assembly govern tissue repair and wound closure in a 3D system [100]. Another microdevice used to measure the forces generated by cells while migrating is the micropillar arrays, which consists of closely and evenly spaced elastic PDMS pillars that enable the measurement of the force based on its deflection [101,102]. By controlling the spatial distribution of the pillar shape and height, it is possible to generate substrate containing heterogeneous rigidity islands. Such a system can then be used to investigate the effect of mechanical heterogeneities on biological function such as endothelial barrier functions in a controlled and reproductive fashion [103]. In an effort to upgrade the design, asymmetric pillars were designed to provide different mechanical properties following the main pillar axis [104,105,106]. The micro-engineered pillars allowed the investigation of how anisotropic mechanical properties can provide migration cues and help steer the cells [105]. Different MEMS were developed and every microfabricated tool has its own characteristics based on the limit range of forces detected, the substrate and its stiffness, and the number of cells seeded to have a result.

## 4. Integrating Microfluidic Devices and Microfabrication to Generate Tumor Models

**Figure 4:**
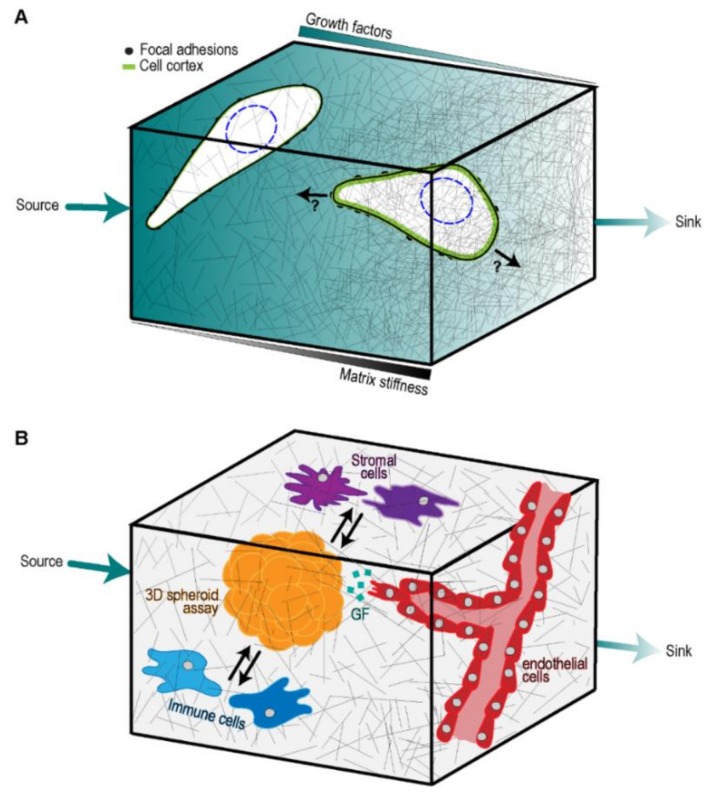
Coupling microfluidic devices with 3D microfabrication as a mimicking model of the TME chemical and physical features. (**A**) 3D microfabricated substrates presenting a matrix stiffness gradient can be coupled with microfluidic device to generate a growth factor gradient. The increase of matrix stiffness as well as GFs promote contractility and cellular migration. This method can be used for conflicting migration cues and determine for example if cell migration in this context is more dictated by chemotaxis or mechanotaxis. (**B**) More complete models mimicking the TME are possible by using co-cultured cells embedded in 3D microfabricated substrates. Co-cultured cells can combine tumor cell 3D spheroids, patient-derived organoids, endothelial cells, stromal cells such as fibroblasts or CAFs, and immune cells such as B lymphocytes. An intercellular crosstalk (black arrow) is established between the spheroid and the others cell types and is managed by direct cell‒cell interactions and indirect communication through different components (e.g., chemokines, cytokines, growth factors). On the one hand, tumor cells secrete different factors, such as VEGF (green square), stimulating the sprouting of vascular endothelial cells. On the other hand, a growth factor gradient can be artificially created and finely controlled to induce a tumor dissemination-like process.

While microfabrication methods allow increasingly accurate depiction of the TME, cell migration is not solely regulated by the ECM. Within tissues, soluble factors can generate gradients that induce polarization and guided cell migration chemotaxis [107]. This is particularly relevant during cancer metastasis where cell migration directly plays a major role. Microfluidic devices have long been used to study these mechanisms by simulating with precision soluble factor gradients [108,109]. Recent efforts have tried to couple these devices with what we have learned from microfabrication of the TME to produce integrated tumor on a chip-like system. Combined approaches provide control over a wide range of TME features, including soluble factors availability, oxygen levels, ECM architecture, and mechanical properties that have allowed inroad into a better understanding of the metastatic process [110,111]. In fact, several different pro-migratory cues and ECM features can all be combined within one device (Figure 4A).

State-of-the-art microfluidic approaches tend to recreate as accurately as possible the complexity of the ECM [112]. Several teams have come up with 3D systems that allow the formation of a precise soluble factor gradient such as oxygen [113], shear stress [114], or tumor-vascular secreted molecules [115]. Type I collagen hydrogel and Matrigel represent the standard 3D scaffolds used within current microfluidic devices [116,117]. Interestingly, tumor cell migration and phenotype are affected by the nature of the scaffold, where a mixture of both collagen and Matrigel allows the highest migration distance and speed [117]. In this study, the collagen and Matrigel mixture were found to be less permeable to the chemoattractant compared to collagen alone. Interestingly, the collagen and Matrigel mixture that allowed maximum migration speed was also stiffer than collagen alone [117]. Furthermore, this concept was extended to the presence of ECM density interfaces, where a growth factor gradient applied from the side of a low-density collagen interface gradient was more efficient in promoting chemotaxis than when applied from the high-density side [117]. More sophisticated than hydrogels, a fabrication method uses electrospun matrices to control the ECM architecture by electrospinning to erect organized polycaprolactone matrix layers. It also provides the possibility to change the matrix material and retrieve the post-experimental matrix [118]. The sophistication of the biomaterial and possible cellular effects requires state-of-the-art equipment in order to assess properly the studied cellular responses. The studies of ECM-dependent cell behavior highlight the importance of considering ECM cell migration properties in a complex environment.

Cell contractility is substrate-dependent, and inversely, cell responses to substrate depend on the contractility state of the cell. Cell contractility is driven by changes in cytoskeletal tensions induced by modifications in the extracellular environment. ECM mechanoproperties influence cell contractility, which in turn will affect cell migration, cell adhesion, and GF signal transduction [15]. Initial simple designs used cast PDMS devices to combine physical confinement with chemoattractant [90,119]. A microfluidic approach emerged using areas of high shear to induce the aggregation of platelets towards block posts, also referred to as force sensors. The contractile force engaged by platelets can be measured through the deflection of the post over time [120]. Furthermore, real time imaging is one of the main features constituting the future of microfluidic approaches. Quantitative polarization microscopy (QPOL) is a new imaging method that can assess and measure cell contractility through cytoskeleton mechanic state at subcellular level. The power of this innovative microscopy is represented by the possible observable range from single cell and 2D monolayer to 3D complex biological samples [28]. Microfluidic devices coupled with a quantitative polarization microscopy (QPOL) in real time could represent a promising association. It could ameliorate the study of cell contractility modulations across different conditions monitored in microfluidic devices such as ECM stiffness, shear stress, and GF gradients.

The role of TME non-malignant cells is significant in tumorigenesis, notably in cell migration. Results have shown cancer cells and stroma cells can increase migration speed, reciprocally acting towards enhancing symbiosis [121]. Interestingly, combining CAFs tumor cells and 3D collagen in a microfluidic chamber resulted in a synergistic amplification of the angiogenic response of endothelial cells, which was not observed when each component was used individually [122]. In a more complex tissue sample, associated tumor spheroids and fibroblast-like cells showed enhanced cytoskeletal tension, epithelial-mesenchymal transition (EMT) biological features, cell motility, spheroid number, and size in a 3D co-culturing microfluidic device [123,124]. Overall, the interaction between tumor cells and stroma cells are essential for tumorigenesis. Replicating these interactions with more complete tumor engineered models will be key to making further inroads into understanding the molecular regulators of metastatic processes (Figure 4B). An interesting technology that could help in increasing the fidelity and lowering the cost of engineered models is the use of bioprinting methods. Most bioprinting approaches use extrusion-based hydrogels that produce large mm scale features [125,126]. However, laser-assisted bioprinting (LAB) is an emerging and promising bioprinting tool that has the potential to achieve the resolution required to produce an engineered TME. Indeed, this technique is already utilized to bioprint large sized vessels [127]. Interestingly, current laser bioprinters have a maximum resolution of 20 µm, which is within the range of possible bioprinting biological entities [126]. Therefore, while still beyond the current systems, this technology could eventually enable the production of small features such small diameter vessels (≤5 µm) and capillaries (3 µm) [128,129], two desirable features in an integrated microfluidic system that are essential to push artificial TME modeling forward.

The major concern in antitumor drug treatment is drug resistance. Microfluidic devices already provide precious help to overcome this problem [130]. Recent investigations have used high throughput screening in microfluidic devices directly with tumor biopsies in order to develop patient specific treatments [131]. A different approach instead combines a multilayered chamber architecture and 3D cell culture to perform combinatory drug screening, with a proof of concept using two standards of care drugs (doxorubicin and paclitaxel) with up to 64 different drug combinations, showcasing the multiplexing ability of this microfluidic system [132]. A future direction will be to see if targeting the ECM physical property can synergize other drug treatments, and microfluidic devices are likely to provide the means to test this possibility. Indeed, there is clinical interest in targeting the ECM. Some drugs indirectly target the ECM, such as anti-CTLA4 (cytotoxic T-lymphocyte-associated protein 4) antibody treatment, which activates immune response [33,133]. Several preclinical drug approaches targeting myeloid cell response to inflammatory cytokines influence MMPs activity and downregulate TGFβ [134], thereby indirectly affecting ECM remodeling within the tumor. Another drug study directly targeted the ECM architecture using specific ECM-destroying enzymes, increasing the permeability of the system so that antitumor treatments can more easily diffuse through the actual tumor for added efficiency [135]. This is an interesting approach as drug delivery within a tumor is indeed a clinical issue [136]. In the light of these results, the success rate of anti-tumor treatments in patients could get better, coupled with in vivo mimicking microfluidic approaches with an improved drug screening. These engineered tissue devices could be used in pre-clinical tests for different cancer subtypes providing critical data to the medical field. Furthermore, the combination of tissue-engineered models and drug screening provides important data about the underlying mechanisms of drug resistance.

The heterogeneity of the cell population present within the tumor provides quite a challenge. The tumor cell population as a whole might not respond to a given treatment as a homogenous population. Here, the flexibility of microfluidic devices brings some interesting possibilities that allow high throughput single cell assays. Indeed, single-cell studies are essential to understand tumor heterogeneity as cancer cells represent a complex group of different cell subpopulations with different properties [137]. Studying tumor cell heterogeneity within the context of the TME at the single cell level with traditional tools is very challenging, while combining single cell analysis with adapted microfluidic devices provides promising results [138,139]. Different methods have been developed in order to undergo single cell analysis based on the combination of optical, magnetic, electrical, and microfluidic approaches [140,141,142]. However, the isolation process removes the environmental contribution from the system. Recently, single-cell co-culture microfluidic methods have been developed to address this issue by including chambers that provide control over the cell‒environment interaction at the single cell level. One such device was used to study different cell sub-population paracrine interactions with a single cell trap with pores that allow paracrine signal to diffuse from a tumor cell population kept in a chamber underneath [143]. Alternatively, a high throughput microfluidic cell pairing device allows the investigation of behaviors of a heterogeneous immune cell population towards different microenvironment conditions via one-to-one cell pairings [144]. Both previous microfluidic devices permit the retrieval of studied cells for single-cell level genomic analysis. These high throughput approaches open the possibility of performing single cell studies within a TME context, thus helping in deciphering the underlying biology and potentially enabling drug screening strategies at a lower cost for pharmacy companies [143,144]. Furthermore, the trapping chamber concept can be expanded to larger components, such as spheroids. In this particular case, one chamber can have a trapped single cell that will grow into a tumor spheroid while a concomitant chamber can contain a stromal cell such as activated fibroblast [145]. Overall, these approaches could in fact allow the different contribution of individual components of the TME to be picked apart to better understand how tumor heterogeneity drives cellular behavior and genetic expression. Our ability to combine these with other single cell techniques, including force measurements and use of advance molecular imaging techniques, to overcome 3D scaffold limitations are key to moving forward. 

## 5. Conclusions and Perspectives 

Traditional in vitro tumor models have long suffered from oversimplification, mainly due to the use of non-physiological substrate with conditions that do not match the disease. Animal models come with their own limitations, either stemming from the difference in the underlying biology of a mouse, or the lack of control over the experimental conditions. Strategies that came from the field of engineering have opened several new lines of investigation. One of the major advantages is the increased control one has over all the possible variables and parameters required in the model. Still, some limitations remain as it is often challenging to perform mechanistic cellular assays within an enclosed, 3D mechanically stable sealed device. Indeed, the ability to combine such a device with proteomics, genomics, and biochemistry will further help in achieving the full potential of these device-based disease models. 

Nevertheless, having access to a more accurate tumor model is certainly an obvious gain for the field. The added complexity provided by in vitro 3D multicellular TME engineered models provides a novel paradigm on which to base our search for improved therapeutics. An important issue that can be addressed is the overall lack of therapeutic target for TMEs, with most drugs currently considered in clinical trial having a severe off target effect given the ubiquitous nature of ECM components [10]. Moreover, since the patient-specific nature of tumors gives rise to a wide distribution of possible mechanical properties, our ability to provide a matching model will certainly lead to improved personalized medicine. Providing an environment that closely mimics the TME makes it more likely that cells will respond in a way that matches their in vivo state. Overall, equipped with enabling models of the tumor condition, research in the field will be able to tackle previously inconceivable mechanisms, ranging from the onset of the disease to terminal metastasis. This will indeed bring an interdisciplinary outlook that will drive research effort, and toward novel clinical targets. The progress made over the last decade has built a strong mechanobiology knowledge base that provides a clear way forward to investigate the importance of the microenvironment during disease progression. Future applications will likely combine multiple approaches, such as microfluidic and force sensors, within the same device. As the field transitions toward more complete and higher throughput TME engineered models, we can expect a big leap in our understanding of tumor biology and drug development from a mechanobiology perspective. These new TME engineered models are likely to become an established high throughput platform for pre-clinical study in the future.

## Figures and Tables

**Table 1:** Comparison of the biological scaffolds and synthetic scaffolds used for cell culture.

Properties	Biological Scaffold	Synthetic Scaffold
Degradability	Degradable with a long-time storage	Better storage stability
Immunogenicity	Poorly immunogenic Antigen content is removed during decellularization	Biocompatibility issue can trigger inflammatory response
Reproducibility	Native architecture is highly preserved (decellularized scaffolds) Batch to batch variation	Very complex architecture High possibility of control
Cell adhesion	Presence of native integrin sites	Lack of specific integrin binding site
Biocompatibility	Depends on the material: Good for native ECM (e.g. collagen, fibrin, etc.) Lower for exogenous biomaterials (e.g. silk, alginate, etc.)	Poor compatibility Cytotoxicity by co-products of degradation

**Table 2:** Characteristics of the most widely used material for microfabrication device for cell biology applications.

Devices	Advantages	Disadvantages
**PDMS based device**	Ease of fabrication Cheap Tunable elasticity Isotropic mechanical properties Excellent material for mold-based device manufacture Selective gas permeability is well suited for microfluidic systems	Modifications restricted to surface properties Possible range of stiffness in the upper range of physiological values >5 kPa
**Collagen based device**	Physiological native ECM Principal ECM component in several type of tumors Fibrillar architecture Controllable pore size 3D architecture is the most representative of in vivo conditions	Costly Hard to control heterogeneity Stiffness and scaffold architecture are hard to decouple Crosslinking process can induce toxicity Not suitable for all cell types Higher stiffnesses are hard to achieve
**PEG based device**	Fully tunable chemistry Precise control of desired architecture and physical properties	Cytotoxicity Low degradability Isotropic physical properties not representative of in vivo

**Table 3:** Reproducing tumor microenvironment (TME) features in 2D versus 3D experimental models.

TME Features	2D Cell Culture		3D Cell Culture
	Traditional (Glass/Plastic Dishes, Plates)	Microfabricated-Engineered Substrate	
**Stiffness**	Non-physiological High stiffness > Mpa-Gpa	Tunable over a physiological range of stiffness	Tunable stiffness High stiffness (>5 kPa) are difficult to achieve using native ECM
**Architecture and spatial organization**	Uniform flat surface	Tunable surface features: Spatial heterogeneities, stiffness gradient	Fully tunable features: Heterogeneities, pore size, matrix density, microarchitecture
**Availability of small molecules**	Free distribution	Free distribution	Material pore size can impede diffusion; Gradients of soluble factors, nutrients and oxygen can form
**Cellular organization**	2D geometry constrains morphogenesis	2D geometry constrains morphogenesis	Free to self-organize in 3D
**Accessibility**	Simplest method, cost can scale with culture conditions	Added complexity Fabrication process can add cost	Increased complexity No standard methods Limited by fabrication cost, ease of use and compatible analytical assays
**Ability to mimic the TME**	-	+	+ +
